# A Novel *NFIX-STAT6* Gene Fusion in Solitary Fibrous Tumor: A Case Report

**DOI:** 10.3390/ijms22147514

**Published:** 2021-07-13

**Authors:** David S. Moura, Juan Díaz-Martín, Silvia Bagué, Ruth Orellana-Fernandez, Ana Sebio, Jose L. Mondaza-Hernandez, Carmen Salguero-Aranda, Federico Rojo, Nadia Hindi, Christopher D. M. Fletcher, Javier Martin-Broto

**Affiliations:** 1 Institute of Biomedicine of Seville (IBiS, CSIC, HUVR, US), 41013 Seville, Spain; dmoura@atbsarc.org (D.S.M.); jdiaz-ibis@us.es (J.D.-M.); csalguero-ibis@us.es (C.S.-A.); 2 Pathology Department, Hospital Virgen del Rocío, 41013 Sevilla, Spain; 3 Centro de Investigación Biomédica en Red del Cáncer (CIBERONC), 28029 Madrid, Spain; 4 Pathology Department–CIBERONC, Sant Pau Hospital, 08041 Barcelona, Spain; sbaguer@santpau.cat (S.B.); ROrellana@santpau.cat (R.O.-F.); 5 Medical Oncology Department, Sant Pau Hospital, 08041 Barcelona, Spain; asebio@santpau.cat; 6 Fundacion Jimenez Diaz University Hospital Health Research Institute (IIS/FJD), 28015 Madrid, Spain; jmondaza@atbsarc.org (J.L.M.-H.); nhindi@atbsarc.org (N.H.); 7 Pathology Department, Fundacion Jimenez Diaz University Hospital, 28040 Madrid, Spain; frojo@fjd.es; 8 Medical Oncology Department, Fundacion Jimenez Diaz University Hospital, 28040 Madrid, Spain; 9 General de Villalba University Hospital, 28400 Madrid, Spain; 10 Department of Pathology, Brigham and Women’s Hospital, Boston, MA 02215, USA; cfletcher@bwh.harvard.edu; 11 Department of Pathology, Harvard Medical School, Boston, MA 02115, USA

**Keywords:** solitary fibrous tumor, gene fusion, STAT6, *NFIX–STAT6*

## Abstract

Solitary fibrous tumor is a rare subtype of soft-tissue sarcoma with a wide spectrum of histopathological features and clinical behaviors, ranging from mildly to highly aggressive tumors. The defining genetic driver alteration is the gene fusion *NAB2–STAT6*, resulting from a paracentric inversion within chromosome 12q, and involving several different exons in each gene. STAT6 (signal transducer and activator of transcription 6) nuclear immunostaining and/or the identification of *NAB2–STAT6* gene fusion is required for the diagnostic confirmation of solitary fibrous tumor. In the present study, a new gene fusion consisting of *Nuclear Factor I X (NFIX),* mapping to 19p13.2 and *STAT6,* mapping to 12q13.3 was identified by targeted RNA-Seq in a 74-year-old female patient diagnosed with a deep-seated solitary fibrous tumor in the pelvis. Histopathologically, the neoplasm did not display nuclear pleomorphism or tumor necrosis and had a low proliferative index. A total of 378 unique reads spanning the *NFIXexon8–STAT6exon2* breakpoint with 55 different start sites were detected in the bioinformatic analysis, which represented 59.5% of the reads intersecting the genomic location on either side of the breakpoint. Targeted RNA-Seq results were validated by RT-PCR/ Sanger sequencing. The identification of a new gene fusion partner for *STAT6* in solitary fibrous tumor opens intriguing new hypotheses to refine the role of *STAT6* in the sarcomatogenesis of this entity.

## 1. Introduction

Solitary fibrous tumor (SFT) is a rare and ubiquitous subtype of soft-tissue sarcoma (STS) that was originally described in pleural tumors by Klemperer and Rabin in 1931 [1,2]. These fibroblastic tumors of mesenchymal origin have an incidence of one new case per million people each year, exhibiting distinct clinical behaviors, from mildly to highly aggressive neoplasms. In line with this, the last WHO classification recommended instead of “typical SFT” or “malignant SFT” terms, the use of risk-stratification models as a better tool to determine prognosis in these tumors [3,4]. The percentage of recurrence into advanced disease can be as high as 40%, and even higher in series with a long follow-up period [5,6,7].

The histopathology of SFT displays a wide range of features, such as a pattern of irregularly arranged spindle to ovoid cells along with other components, staghorn-shaped blood vessels and a prominent collagenous stroma. The pathological spectrum can vary from highly fibrotic with scanty cells to highly cellular, as well as the dedifferentiated variants with an abrupt transition from a low-grade area to a high-grade sarcoma. This latter subtype is inherently associated with worse prognoses.

The identification of intrachromosomal *NAB2–STAT6* gene fusion as a pathognomonic molecular feature of SFT in 2013 [8,9,10] contributed to unequivocally unify under the unique term SFT other supposed distinct conditions, such as hemangiopericytoma and giant cell angiofibroma. In fact, for the purpose of definitive diagnostic confirmation, nuclear staining for STAT6 or the demonstration of *NAB2–STAT6* gene fusion is required. As SFT can mimic many mesenchymal (and non-mesenchymal) tumors, the availability of these tools has become crucial for an accurate diagnosis of SFT [11]. The genomic fusions between *NGFI-A-binding protein 2* (*NAB2*) and *signal transducer and activator of transcription 6* (*STAT6*) can involve several exons in both genes, but they always produce a protein transcript without at least one repressor domain from *NAB2*, which is replaced by an activation domain of *STAT6*. Most investigators have attributed to *NAB2* gene dysfunction the key genomic driver of the fusion transcript *NAB2–STAT6*. The lack of transcriptional corepression exerted on *EGR1* by a *NAB2* truncated gene would explain the increase in EGR1-mediated transcription in SFT [8,9].

In the report presented here, we describe a tumor lesion that exhibits histological features of SFT, exhibits nuclear positivity for STAT6 and carries a gene fusion, affecting *STAT6* and the *NFIX* gene, another partner gene that is different than *NAB2*. The discovery of a new fusion partner in SFT opens intriguing new ways to refine the role of *STAT6* in the sarcomatogenesis of this entity.

## 2. Results

### 2.1. Clinical and Pathological Features

A 74-year-old female, diagnosed in 2002 (at the age of 60 years old) with a deep-seated SFT in the pelvis, was sequenced for gene fusion detection with the Archer FusionPlex^TM^ Sarcoma Panel, as a pathological central review procedure (at dept. Pathology, Hospital Virgen del Rocío) for enrollment in the GEIS-32 clinical trial (ClinicalTrials.gov: NCT02066285; EudraCT number: 2013-005456-15).

The patient was diagnosed in 2002 and treated with primary radical surgery. In 2009, she presented a local relapse and was treated with surgery, which included a bladder partial resection as well as a hysterectomy and bilateral oophorectomy. In 2010, she developed a new pelvic relapse, which was treated with an R1 resection. In subsequent scans, new evidence of disease was seen progressing slowly until June 2014, when a debulking surgery was performed due to symptomatic progression.

The patient was enrolled in the GEIS 32 clinical trial in May 2016 and started therapy with pazopanib 800 mg/day, achieving stable disease by CHOI and RECIST 1.1 evaluations, but treatment was interrupted due to grade 3 hepatic toxicity. Progression-free survival was 3.37 months. Post-protocol treatments included sunitinib, from October 2016 to May 2017, which achieved a partial response that led to a new surgery performed in June 2017. The patient was followed up without evidence of disease until January 2019, with slow progression in subsequent CT scans. In December 2019, a new surgical procedure was performed and since then, the patient has been under close surveillance for slowly progressing disease located in the pelvis. At the time of the last follow up in January 2021, the patient was alive and asymptomatic.

**Figure 1:**
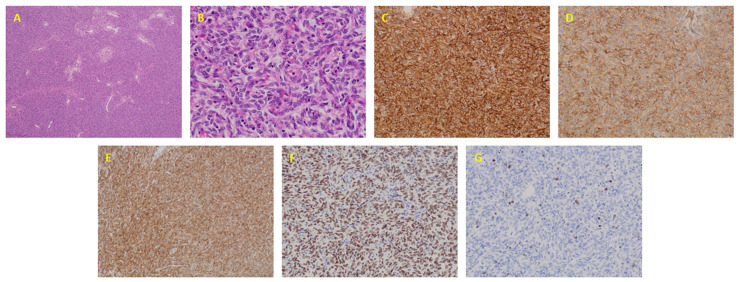
Pathological characterization of solitary fibrous tumor case. (**A**) Hypercellular spindle cell neoplasm with hemangiopericytoid vascular pattern (4×); (**B**) Monotonous spindle to ovoid cells, without nuclear pleomorphism (40×); (**C**) Diffuse expression of CD34 (20×); (**D**) Heterogeneous expression of CD99 (20×); (**E**) Diffuse expression of bcl-2 (20×); (**F**) Diffuse and strong nuclear expression of STAT6 (20×) and (**G**) Ki-67 proliferative index (20×).

The local pathology report of the 2014 excisional biopsy described a hypercellular spindle cell neoplasm with hemangiopericytoid vascular pattern, with monotonous spindle to ovoid cells, without nuclear pleomorphism or tumor necrosis and with a low proliferative index. Complementary studies showed diffuse immunostaining for CD34, heterogeneous expression for CD99, diffuse expression of bcl-2 and diffuse and strong nuclear expression of STAT6; proliferative rate (Ki67) less than 2% (Figure 1).

### 2.2. Fusion Description

**Figure 2:**
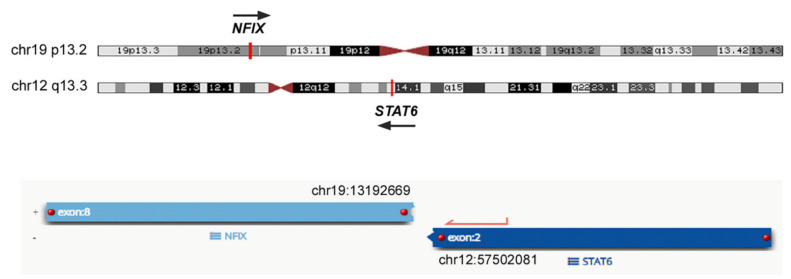
Genomic location on either side of the breakpoint of the *NFIXex8–STAT6ex2* gene fusion.

The tumor excised in 2014 was sequenced for gene fusion and breakpoint variant detection with the Archer FusionPlex^TM^ Sarcoma Panel (Boulder, CO, USA). The results from FFPE tumor sample sequencing reveal a novel fusion transcript involving *Nuclear Factor I X* (*NFIX*), mapping to 19p13.2 and *STAT6*, mapping to 12q13.3. Total sample reads were 1,140,402. The bioinformatics analysis detected 378 unique reads spanning the *NFIX_ex8_–STAT6_ex2_* breakpoint with 55 different start sites, representing 59.5% of the reads intersecting the genomic location on either side of the breakpoint (Figure 2). This new fusion gene was detected in a non-treated surgical specimen (after the fourth surgery). No other gene fusion transcripts were detected in the same sample.

**Figure 3:**
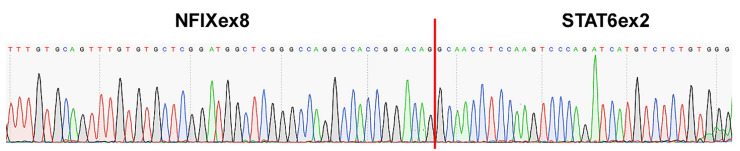
Sanger sequencing validation of *NFIX_ex8_–STAT6_ex2_* gene fusion.

The new *NFIX–STAT6* gene fusion and its specific breakpoint were validated using a set of primers for RT-PCR. Sanger sequencing was performed on the amplicon product and the sequence obtained was identical to the one founded by next generation sequencing (NGS). The breakpoint involved the exon 8 of *NFIX* and the exon 2 of *STAT6* (Figure 3).

**Figure 4:**
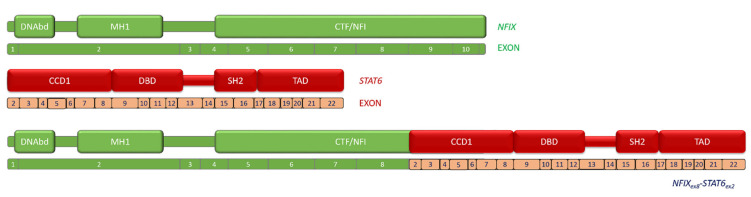
Schematic representation of *NFIX–STAT6* fusion. DNAbd: N-terminal DNA-binding domain; MH1: MAD homology 1 domain; CTF/ NFI: CAAT-box transcription factor–nuclear factor I domain; CCD1: Coiled-coil domain 1; DBD: DNA-binding domain; SH2: Src homology 2; and TAD: transcriptional activator domain.

The in-frame breakpoints of the two genes occurred at nucleotide 1332 for *NFIX* (NM_001271043.2) and nucleotide 254 for *STAT6* (NM_001178078.2). The novel fusion transcript maintained both the MAD homology 1 (MH1) domain and the N-terminal DNA-binding (DNAbd) domain in the N-terminus of NFIX and a larger part of its CAAT-box transcription factor–nuclear factor I (CTF-NFI) domain. The complete STAT6 protein is present in the *NFIX–STAT6* fusion (Figure 4).

## 3. Discussion

In this report, a novel fusion gene, *NFIX–STAT6*, is described in a patient diagnosed with a tumor that histopathologically and by immunohistochemistry exhibits characteristic traits of SFT. To the best of our knowledge, this is the first time ever that this fusion has been detected in cancer or any other disease, and is the first time that a different partner for *STAT6*, apart from *NAB2*, has been identified in SFT. This new fusion gene would suggest that *STAT6* is also relevant in the potential mechanisms of SFT oncogenesis and progression, independently of the potential pivotal role of *NAB2* as the driver of the tumorigenic processes of SFT within the *NAB2–STAT6* fusion gene [9,10].

The majority of these fusions replace the chromodomain helicase DNA-binding protein 4 (CHD4) interacting domain (CID) in the C-terminus of *NAB2*, but they can also affect the N-terminus, replacing the *NAB2* conserved domain 2 (NCD2). These two domains (i.e., CID and NCD2) are important for transcriptional repression and as a consequence of their lack of activity, and since early growth response 1 (*EGR1*)-interacting NCD1 is always present in the fusion protein, the EGR signaling pathway is normally positively dysregulated in SFT [9,10]. However, even if the key oncogenic role of *NAB2–STAT6* may be related to the lack of repressive activity of *NAB2* upon its targets, *STAT6* dysfunction could also play an important part in SFT development. In fact, *STAT6* seems to play a significant role in transcription and inflammation [12,13], processes linked to cancer development and progression [14,15,16,17,18].

According to *NAB2–STAT6* fusion breakpoints, several different variants have been described; the two most frequent variants are the *NAB2exon4–STAT6 exon2* (*NAB2_ex4_–STAT6_ex2_*) and the *NAB2exon6–STAT6exon16/17* (*NAB2_ex6_–STAT6_ex16/17_*) [19]. The *NAB2_ex4_–STAT6_ex2_* fusion lacks almost entirely the CID domain of *NAB2*, while *STAT6* is almost complete. The *NAB2_ex6_–STAT6_ex16/17_* lacks only the CID-exon 7 of *NAB2*, whereas *STAT6* only keeps the last 5/6 exons, which includes part of the Src homology 2 domain (SH2) and the transcriptional activator domain (TAD). The SFTs harboring the *NAB2_ex4_–STAT6_ex2_* variant are typically located in the thoracic cavity, have significantly larger tumor diameter (median 10 cm), exhibit the appearance of predominantly fibrotic and paucicellular features and the patients are normally older compared to the patients with other breakpoint variants. On the other hand, SFTs harboring the *NAB2_ex6_–STAT6_ex16/17_* variant are commonly founded in the pelvis, meninges or extremities, have small tumor diameter (median 4.3 cm), show higher tumor cellularity compared to the previous fusion breakpoint variant and the patients are younger than those with the *NAB2_ex4_–STAT6_ex2_* fusion [19,20]. However, and despite the more frequent association of the *NAB2_ex4_–STAT6_ex2_* variant with less aggressive SFT, there is no unequivocal significant prognostic correlation between fusion breakpoints and survival [21]. Curiously, the fusion gene variant with the hardly truncated *NAB2* gene, the *NAB2_ex6_–STAT6_ex16/17_* variant, is associated with SFT features of higher aggressiveness. Besides *NAB2–STAT6*, no other fusion genes had been described in SFT until now.

STAT6 is a member of the signal transducer and activator of transcription family of proteins that is activated, in response to IL-4 or IL-13, by phosphorylation of tyrosine residues. These post-translational modifications promote homodimerization or heterotrimerization with other transcription factors, which translocate into the nucleus to initiate transcription [22]. Even when it is also recognized that non-phosphorylated STAT6 can be transcriptionally active [23], the STAT6 nuclear trafficking is tightly regulated. STAT6 is a predominantly cytoplasmic protein in a variety of cell types, as well as in tumors other than SFT [24]. The nuclear relocation of STAT6, which NAB2 localizes to the nucleus, with the transcript *NAB2–STAT6,* could lead to the activation of target genes of *STAT6*.

The pathway (IL-4/ STAT6) is important, for example, for type 2 T helper (Th2) cells’ development that can induce the differentiation of macrophages to an M2 phenotype [25,26]. Of note, M2 polarized macrophages seem to have an important prognostic role in SFT in two recent publications taken from a prospective multicenter phase II clinical trial of pazopanib for the treatment of advanced SFT [27,28]. Patients with high expression of *CD209* had worse prognoses in terms of PFS and overall survival (OS) in the typical SFT cohort, while *ISG15* high expression was associated with worse survival in the malignant/dedifferentiated SFT cohort. *CD209* encodes a transmembrane receptor on the surface of both dendritic cells and macrophages, and it was defined as a marker of M2 polarized macrophages [29] and *ISG15*, an interferon-stimulated gene with a role in protein degradation and stemness, which seems to be involved in cell migration and CD8^+^ T cell immunosuppression by inducing M2-like macrophages [30]. Accordingly, it is plausible that STAT6-dependent regulation of macrophage polarization towards an M2-like phenotype may play an important role in SFT tumorigenesis and tumor progression.

Of note, the *NFIX–STAT6* gene fusion maintained the complete STAT6 protein (exon 2 to 22), supporting the relevance of this transcription factor in the oncogenic processes driven by the chimeric RNA.

The *NFIX* gene is a member of the NFI (nuclear factor 1) transcription factor family, along with three other related genes involved in the regulation of stem cell biology during development. Initially, NFIX was identified as a transcription factor for fetal myogenesis; however, it is now recognized for its involvement in neural development and hematopoiesis [31]. *NFIX* gene expression has been shown to be significantly lower in some tumors (medulloblastoma, colorectal cancer or glioma), where it has been implicated as a tumor suppressor gene [32]. We can speculate that the nuclear protein NFIX could have lost its repressor function in some mesenchymal stem cells in the transcript, and similarly to NAB2, the transcript would retain STAT6 in the nucleus.

Gene fusions involving members of the NFI family have been reported in different types of cancer. However, functional relevance remains to be determined for most of them [33]. A recent report on secretory carcinoma of the skin described a novel *NFIX–PKN1* translocation, but again, its oncogenic properties are not known [34], thus reducing our knowledge regarding its potential oncogenicity.

Finally, as besides *NAB2–STAT6,* no other fusion genes have been described in SFT, the newly detected *NFIX–STAT6* fusion opens the inquiry to concede more relevance than just a secondary role to the *STAT6* gene. This implies the opportunity for a wider perspective of new potential targets, such as macrophage implication in SFT progression for instance. This new discovery also spotlights the *NFIX* gene for deeper investigation of plausible signaling pathways related to SFT pathogenesis.

## 4. Material and Methods

### 4.1. Targeted RNA-Seq Library Preparation and Sequencing

Total nucleic acid was extracted with an Agencourt FormaPure kit (A33341; Beckman Coulter, Indianapolis, IN, USA), according to the manufacturers’ protocol. The extracted RNA was quantified using the Qubit^®^ RNA HS Assay kit in combination with a Qubit^®^ fluorometer (Q32852; Thermo Fisher Scientific, Waltham, MA, USA). Targeted library was prepared using the Archer™ FusionPlex™ Sarcoma Panel (SK0082; ArcherDX, Boulder, CO, USA) based on a targeted enrichment method named anchored multiplex PCR (AMP). A total of 200 ng of RNA was used for library preparation. The Archer™ FusionPlex™ Sarcoma Panel simultaneously detects and identifies fusions of 26 genes associated with soft-tissue sarcoma, without prior knowledge of the fusion partners or the specific breakpoints. In summary, RNA was reverse transcribed using random primers, first strand cDNA was synthesized and RNA quality was assessed using the Archer PreSeq RNA QC assay. After second strand cDNA synthesis, end repair, A-tailing and adapter ligation, cDNA was amplified by two rounds of nested PCR using gene-specific primers. KAPA Library Quantification Kit (KK4824; KAPA Biosystems, Wilmington, MA, USA) was used to quantify the final libraries. These libraries were sequenced on an Illumina MiSeq with MiSeq 300v2 reagents (MS-102-2002; Illumina, San Diego, CA, USA) for paired-end reads, 150 base pair read and dual index reads. FASTQ files were analyzed using Archer analysis pipeline version 6.0.3.2 and genome assembly GRCh37/hg19 as reference.

### 4.2. RT-PCR and Sanger Sequencing

Primers flanking the breakpoint sequence were designed with Primer3Plus [35], according to the reads from RNA sequencing. First, 450 ng of RNA was retro-transcribed using High-Capacity cDNA Reverse Transcription Kit (Thermo Fisher Scientific). PCR was performed with Q5^®^ Hot Start High-Fidelity 2X Master Mix (New England Biolabs, MA, USA) and the specific primers. The PCR products were analyzed by gel electrophoresis and purified using QIAquick PCR Purification Kit (Qiagen, Germany). Direct Sanger sequencing of PCR products was performed using BigDye Terminator v3.1 chemistry. Primer forward: AGCAGTCGAGCCCGTATTTC and primer reverse: ACCAGACCCCACAGAGACAT (product length 163bp).

### 4.3. Immunohistochemistry

The resected specimen was fixed in 10% neutral buffered formalin. Paraffin sections were stained with hematoxylin and eosin for routine histology. Immunohistochemical studies were performed on paraffin-embedded tissue sections using an automated OMNIS immunostainer (Agilent Dako, CA, USA), followed by antibody detection using the Dako EnVision+System and 3,3′-diaminobenzidine as a chromogen. The primary antibodies, clone, dilutions and sources used in this study are listed in Table 1. Appropriate positive and negative tissue controls were used throughout.

## Figures and Tables

**Table 1:** Immunohistochemical reagents.

Antigen	Clone	Dilution	Source
Bcl-2	124	Ready to use	Agilent
CD34	QBEnd 10	Ready to use	Agilent
CD99	12E7	Ready to use	Agilent
STAT6	Polyclonal	1:50	Gennova

## Data Availability

Not applicable.
